# Antiferromagnetic topological insulator with selectively gapped Dirac cones

**DOI:** 10.1038/s41467-023-42782-6

**Published:** 2023-11-17

**Authors:** A. Honma, D. Takane, S. Souma, K. Yamauchi, Y. Wang, K. Nakayama, K. Sugawara, M. Kitamura, K. Horiba, H. Kumigashira, K. Tanaka, T. K. Kim, C. Cacho, T. Oguchi, T. Takahashi, Yoichi Ando, T. Sato

**Affiliations:** 1 Department of Physics, Graduate School of Science, Tohoku University, Sendai, 980-8578 Japan; 2 Center for Science and Innovation in Spintronics (CSIS), Tohoku University, Sendai, 980-8577 Japan; 3 Advanced Institute for Materials Research (WPI-AIMR), Tohoku University, Sendai, 980-8577 Japan; 4 Center for Spintronics Research Network (CSRN), Osaka University, Toyonaka, Osaka 560–8531 Japan; 5 Institute of Physics II, University of Cologne, Köln, 50937 Germany; 6 Precursory Research for Embryonic Science and Technology (PRESTO), Japan Science and Technology Agency (JST), Tokyo, 102-0076 Japan; 7 Institute of Materials Structure Science, High Energy Accelerator Research Organization (KEK), Tsukuba, Ibaraki 305-0801 Japan; 8 National Institutes for Quantum Science and Technology (QST), Sendai, 980-8579 Japan; 9 Institute of Multidisciplinary Research for Advanced Materials (IMRAM), Tohoku University, Sendai, 980-8577 Japan; 10 UVSOR Synchrotron Facility, Institute for Molecular Science, Okazaki, 444-8585 Japan; 11 Diamond Light Source, Harwell Science and Innovation Campus, Didcot, Oxfordshire OX11 0QX UK; 12 International Center for Synchrotron Radiation Innov1ation Smart (SRIS), Tohoku University, Sendai, 980-8577 Japan

**Keywords:** Topological insulators, Electronic properties and materials

## Abstract

Antiferromagnetic (AF) topological materials offer a fertile ground to explore a variety of quantum phenomena such as axion magnetoelectric dynamics and chiral Majorana fermions. To realize such intriguing states, it is essential to establish a direct link between electronic states and topology in the AF phase, whereas this has been challenging because of the lack of a suitable materials platform. Here we report the experimental realization of the AF topological-insulator phase in NdBi. By using micro-focused angle-resolved photoemission spectroscopy, we discovered contrasting surface electronic states for two types of AF domains; the surface having the out-of-plane component in the AF-ordering vector displays Dirac-cone states with a gigantic energy gap, whereas the surface parallel to the AF-ordering vector hosts gapless Dirac states despite the time-reversal-symmetry breaking. The present results establish an essential role of combined symmetry to protect massless Dirac fermions under the presence of AF order and widen opportunities to realize exotic phenomena utilizing AF topological materials.

## Abstract (web summary)

Antiferromagnetic topological materials have attracted attention recently due to their unique quantum properties and application potential. Here the authors establish an antiferromagnetic topological insulator in NdBi and demonstrate gapped and gapless surface states in two different magnetic domains.

## Introduction

To realize exotic quantum states in the topological insulator (TI), it is often necessary to break the time-reversal symmetry by introducing ferromagnetism into the crystal^1–4^, as highlighted by the observation of quantum anomalous Hall effect^5–7^. As a natural extension, antiferromagnetic (AF) TIs are recently attracting particular attention because it is expected to show exotic properties^1,8–17^, such as quantized magneto-electric effect accompanied by surface Hall conductivity^1,8,18,19^, gigantic magneto-optical responses by AF fluctuations^9,20^, and dynamic axion field useful for detecting dark-matter axions^21^. Also, AF TI is a useful platform applicable to spintronic devices owing to the ultrafast spin response and zero stray magnetic field^22,23^. To provide a pathway toward realizing exotic properties associated with antiferromagnetism and topology, it is essential to establish a new AF topological material, in particular, AF TI.

**Fig. 1:**
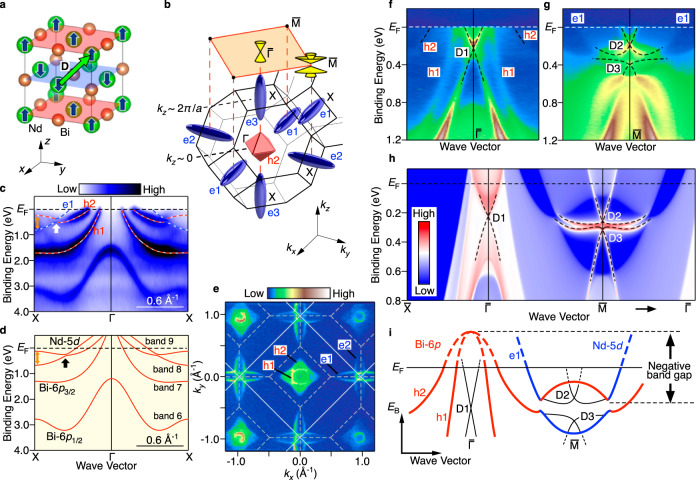
Band structure of NdBi in the paramagnetic phase. **a** Rock-salt crystal structure of NdBi with spin orientation in the antiferromagnetic (AF) phase. Translation **D** vector is also indicated by the green arrow. **b** Schematic Fermi surface (FS) and bulk fcc Brillouin zone (BZ) of NdBi, together with the surface BZ projected onto the (001) plane (orange rectangle) and Dirac-cone surface state (SS) at the $\bar{\Gamma }$ and $\bar{{{{{{\rm{M}}}}}}}$ points. **c** Angle-resolved photoemission spectroscopy (ARPES) intensity at *T* = 40 K measured along the ΓX cut (*k*_*z*_ ~ 0) of bulk BZ with soft X-ray photons of *hv* = 515 eV. Red and blue dashed curves highlight the band dispersion for the Bi-6*p* (h1, h2) and Nd-5*d* (e1) orbital. The spin-orbit gap at the intersection of these bands and the band inversion at the X point are indicated by white and orange arrows, respectively. **d** Corresponding calculated bulk band structure in the paramagnetic (PM) state. The label of bands (6–9) is also indicated. **e** ARPES-intensity mapping at *E*_F_ as a function of *k*_*x*_ and *k*_*y*_ at *T* = 35 K measured at *hν* = 60 eV. **f** ARPES intensity around the $\bar{\Gamma }$ point of surface BZ measured at *hν* = 75 eV. **g** Same as (**f**) but measured around the $\bar{{{{{{\rm{M}}}}}}}$ point at *hν* = 60 eV. Black dashed curves in (**f**, **g**) are guides for the eyes to trace the surface band dispersions. **h** Calculated surface spectral weight along the $\bar{\Gamma }\bar{{{{{{\rm{X}}}}}}}$ and $\bar{\Gamma }\bar{{{{{{\rm{M}}}}}}}$ cuts projected onto the (001) plane, obtained with the Green-function method for a semi-infinite slab of NdBi in the PM phase. Black dashed curves trace the band dispersion of D1–D3 SS. **i** Schematic band structure of NdBi in the PM phase. D1–D3 indicated by thin black curves represent topological SS, whereas red and blue curves are bulk Bi-6*p* bands forming hole pockets (h1 and h2) at $\bar{\Gamma }$ and Nd-5*d* band forming an electron pocket (e1) at $\bar{{{{{{\rm{M}}}}}}}$, respectively.

Prediction of AF TI was first made by Mong et al. in 2010 in their tight-binding model for the NaCl lattice with type-I anfiferromagnetism^2^. While the time-reversal symmetry (*Θ*) is broken in the AF phase, the combined symmetry (*S* = *ΘT*_D_), where *T*_D_ represents the translation by the **D** vector that inverts the spin direction (Fig. 1a), is preserved^8,12,13^. Materials having this *S* symmetry are characterized by the *Z*_2_ topological invariant as in the case of time-reversal-invariant TIs^8,12,13^ and have been predicted to show a weak-TI-like behavior wherein the Dirac-cone SS is protected for the crystal plane parallel to the **D** vector, otherwise gapped. Thus, the AF TI has a unique experimental advantage distinct from strong TIs and ferromagnetic TIs; namely, the controllability of Dirac-cone SS through the manipulation of the **D** vector. This advantage manifests as intriguing characteristics of surface Dirac fermions; namely, the Dirac mass is strongly anisotropic and depends on the configuration of the AF structure, distinct from so far established strong 3D TIs and topological semimetals. The protection by the *S* symmetry in AF TI is a key ingredient to realize exotic properties^8–11,15,19,24^. It is thus essential to spectroscopically establish AF TI by distinguishing surfaces protecting/breaking *S* symmetry.

As a candidate of AF TIs protected by *S* symmetry, some materials such as MnBi_2*n*_Te_3*n*+1_ (MBT), EuIn_2_As_2_, and EuCd_2_As_2_ have been theoretically predicted^14,15,25–27^. The transport property that supports the topological nature of AF TIs such as the quantum anomalous Hall effect in MBT (*n* = 1) has been intensively investigated^8,14,28^, and various attempts to examine the associated magnetic gap of the Dirac-cone state in magnetic TI candidates have been made^14,29–33^. However, these materials have a layered structure and spectroscopies can access only a single surface parallel to the layer, making it difficult to obtain deep insights into the relationship between the AF order and topological SS. Also, in the case of MBT, there exists a fierce controversy on the magnitude and gapless/gapful nature of the Dirac-cone SS, which is not settled at the moment^34–40^. In this regard, the rare-earth monopnictide NdBi that exhibits type-I AF order (Fig. 1a) below Néel temperature of *T*_N_ = 24 K is an excellent platform from the spectroscopic viewpoint, because (i) the cubic crystal structure is suited to access surfaces with different AF domains^41^ and (ii) the large magnetic moment (3*μ*_B_) and large chemical ratio (50%) of Nd ions may lead to a large magnetic Dirac gap. This provides us a precious opportunity to investigate the topological property unique to the AF TI, although the bulk semimetallic nature of monopnictides may hinder the quantum transport expected for AF TIs. A recent angle-resolved photoemission spectroscopy (ARPES) study on NdBi reported an unusual SS in the AF phase^42^, making this material more interesting to study the entanglement between the AF order and electronic states (note that such SS was recently proposed to be a topologically trivial state and unlikely to be directly related to the AF TI properties^43^). Here, by utilizing the AF-domain-selective micro-focused ARPES, we established the electronic states of both *S*-preserving and *S*-broken surfaces with different Dirac-electron characteristics in NdBi.

## Results and discussion

### *Z*_2_ topology in the paramagnetic phase

First, we present the overall band structure of NdBi in the paramagnetic (PM) phase. Rare-earth monopnictide (RX_p_) is a semimetal characterized by two hole pockets at the $\bar{\Gamma }$ point and electron pockets at the X point in the face-centered cubic (fcc) Brillouin zone (BZ) (Fig. 1b; refs. ^44,45^). Bulk-sensitive ARPES with soft-X-ray photons together with first-principles band-structure calculations signifies bulk bands forming these pockets (Fig. 1c, d) together with the signature of bulk band inversion (for details, see Supplementary Notes 1 and 2). The Fermi-surface (FS) mapping in Fig. 1e obtained with vacuum ultraviolet (VUV) photons reveals corresponding features in the surface BZ. At the $\bar{\Gamma }$ point, we found inner square-like (h1) and outer diamond-like (h2) pockets which originate from the Bi-6*p* hole bands (Fig. 1d). In Fig. 1e, one can also recognize vertically (e2) and horizontally (e1) elongated pockets at the $\bar{{{{{{\rm{M}}}}}}}$ point originating from Nd-5*d* bands at X points of different *k*_*z*_’s (*k*_*z*_ = 0 and 2*π*/*a*, respectively; Fig. 1b) due to the strong *k*_*z*_ broadening of VUV photons^45–47^. The utilization of surface-sensitive VUV photons enables us to visualize the topological SS. One can identify in Fig. 1f a Dirac-cone band (called D1)^47–50^ around the $\bar{\Gamma }$ point. While there is a single Dirac cone located at the binding energy *E*_B_ of ~0.2 eV (which we call the Dirac-point energy *E*_DP_) at the $\bar{\Gamma }$ point, the ARPES intensity across the $\bar{{{{{{\rm{M}}}}}}}$ point in Fig. 1g signifies double Dirac-cone bands with *E*_DP_ ~0.2 and ~0.4 eV (refs. ^47–49,51^), called here D2 and D3, respectively. All these D1–D3 bands are confirmed to be of surface origin from their *hv*-independent energy position (for details, see Supplementary Note 3). The difference in the number of Dirac cones at two different time-reversal-invariant momenta (TRIM) at the surface corresponds to the difference in the number of band inversion in the bulk, namely, a single bulk X point is projected onto the $\bar{\Gamma }$ point whereas two inequivalent bulk X points onto the $\bar{{{{{{\rm{M}}}}}}}$ point (see Fig. 1b). Odd numbers of Dirac cones in total suggest that NdBi in the PM phase is a *Z*_2_ TI with a negative band gap (Fig. 1i), consistent with the parity analysis of the bulk-band structure which suggests the strong TI nature with (*v*_0_; *v*_1_, *v*_2_, *v*_3_) = (1; 0, 0, 0); for details, see Supplementary Table S1. Our surface-projection calculations for the PM phase also reproduce the D1–D3 Dirac-cone states (Fig. 1h), consistent with the band picture obtained from the experiment (Fig. 1i; for details, see Supplementary Note 2).

### AF-induced reconstruction of the Dirac-cone SS

**Fig. 2:**
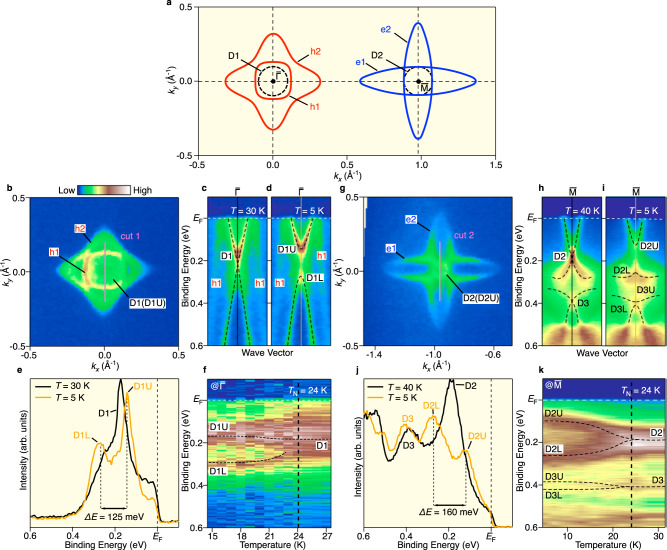
Observation of massive Dirac-cone bands in the AF phase. **a** Schematic FS of NdBi projected onto the surface BZ. e1 and e2 (blue curves) represent electron pockets elongated along *k*_*x*_ and *k*_*y*_, respectively, and are located at different X points in the bulk BZ. h1 and h2 (red curves) represent inner and outer hole pockets, respectively. **b** FS mapping around the $\bar{\Gamma }$ point in the AF phase (*T* = 5 K) measured at *hν* = 60 eV. **c**, **d** ARPES intensity around the $\bar{\Gamma }$ point in the PM phase (*T* = 30 K) and the AF phase (*T* = 5 K), respectively, measured at *hν* = 75 eV. **e** EDCs at the $\bar{\Gamma }$ point at *T* = 5 K (orange curve) and 30 K (black curve). Dashed lines are a guide for the eyes to trace the upper and lower D1 (D1U and D1L). **f** Temperature dependence of ARPES intensity at the $\bar{\Gamma }$ point. Dashed curves are a guide for the eyes to highlight the gap opening across *T*_N_ for D1. **g**–**i** Same as (**b**–**d**) but around the $\bar{{{{{{\rm{M}}}}}}}$ point obtained at *hν* = 60 eV. Dashed curves in (**h**, **i**) are a guide for the eyes to trace the band dispersion of D2 and D3 as well as their lower and upper branches. **j**, **k** Same as (**e**, **f**) but measured at the $\bar{{{{{{\rm{M}}}}}}}$ point. Dashed curves in (**k**) are a guide for the eyes to highlight the gap opening across *T*_N_ for D2 and D3.

Next, we show how the AF order influences the topological nature of NdBi. As summarized in Fig. 2a, the FS topology in the PM phase projected onto the surface BZ is characterized by the h1 and h2 hole pockets at $\bar{\Gamma }$, the D1 SS, the elongated e1 and e2 pockets at $\bar{{{{{{\rm{M}}}}}}}$, and the D2 SS. As shown by the FS mapping around the $\bar{\Gamma }$ point at *T* = 5 K in Fig. 2b, all the pockets observed in the PM phase (h2, h1, and D1) are also resolved in the AF phase. A comparison of the band dispersion across the $\bar{\Gamma }$ point between the PM (Fig. 2c; *T* = 30 K) and AF phases (Fig. 2d; *T* = 5 K) shows that the D1 band is kept observed at both temperatures, indicating that the bulk-band inversion is preserved in the AF phase; however, a careful look at the D1 band reveals that the V-shaped upper branch (D1U) moves upward in the AF phase relative to that in the PM phase, while the Λ-shaped lower branch (D1L) moves downward, resulting in the opening of a Dirac gap associated with time-reversal-symmetry breaking due to the AF order. This gap is confirmed in more careful analyses of the energy distribution curve (EDC) at the $\bar{\Gamma }$ point (Fig. 2e) which signifies a double peak at *T* = 5 K as opposed to a single peak at *T* = 30 K, and its AF origin is also supported by the detailed temperature-dependent ARPES-intensity variation at the $\bar{\Gamma }$ point (Fig. 2f; for details, see Supplementary Note 4 and Supplementary Fig. 5). The magnitude of the Dirac gap at $\bar{\Gamma }$ estimated from the EDC is 125 ± 5 meV. This value is unexpectedly large despite the zero net magnetization and low *T*_N_ (24 K) in NdBi and may be related to the effectively large local exchange field although the exact mechanism is unclear at the moment (for a more detailed discussion, see Supplementary Note 4). Such a sizable Dirac gap that is directly linked to the AF order is in sharp contrast to that of an intrinsic magnetic TI candidate MBT where the Dirac gap is elusive^14,29–40^.

Now we turn our attention to the AF-induced reconstruction of the other Dirac-cone bands located at the $\bar{{{{{{\rm{M}}}}}}}$ point. Although the spectral features are complicated by the presence of two Dirac-cone bands D2 and D3 in the PM phase (Fig. 2h), these bands still survive in the AF phase (Fig. 2i), as also seen in the FS mapping in the AF phase in Fig. 2g, where a small circular pocket originating from the upper branch of the D2 band (D2U) is observed inside the elongated bulk electron pockets, e1 and e2. The gapless X-shaped dispersion of the D2 band in the PM phase (Fig. 2h) turns into the upper (D2U) and lower (D2L) bands separated by a Dirac gap in the AF phase (Fig. 2i). This is also evident from the EDCs at the $\bar{{{{{{\rm{M}}}}}}}$ point in Fig. 2j where a peak located at *E*_B_ = 0.19 eV at *T* = 40 K splits into two peaks at 0.12 and 0.28 eV at *T* = 5 K, exhibiting a Dirac gap of 160 ± 5 meV. The Dirac gap opening for the D2 and D3 bands in the AF phase is also supported by our slab calculations for the AF phase (for details, see Supplementary Fig. 2).

To validate the AF origin of the observed spectral change, we have performed temperature-dependent ARPES measurements across *T*_N_. The ARPES intensity at the $\bar{{{{{{\rm{M}}}}}}}$ point plotted against temperature in Fig. 2k signifies no discernible change in the intensity profile above *T*_N_, as evident from the *T*-invariant *E*_B_ position of the D2 band. On the other hand, the D2 band splits into two bands as soon as the sample is cooled down below *T*_N_. On lowering the temperature, the splitting is gradually enhanced and almost saturated below *T* = 15 K. This unambiguously demonstrates the AF origin of the Dirac gap. We note that the splitting of the D3 band was not clearly observed because of its weak intensity; the gap is likely much smaller than that of the D2 band (<40 meV). This trend is also recognized in the slab calculation shown in Supplementary Fig. 2.

### Domain-selective electronic states in the AF phase

**Fig. 3:**
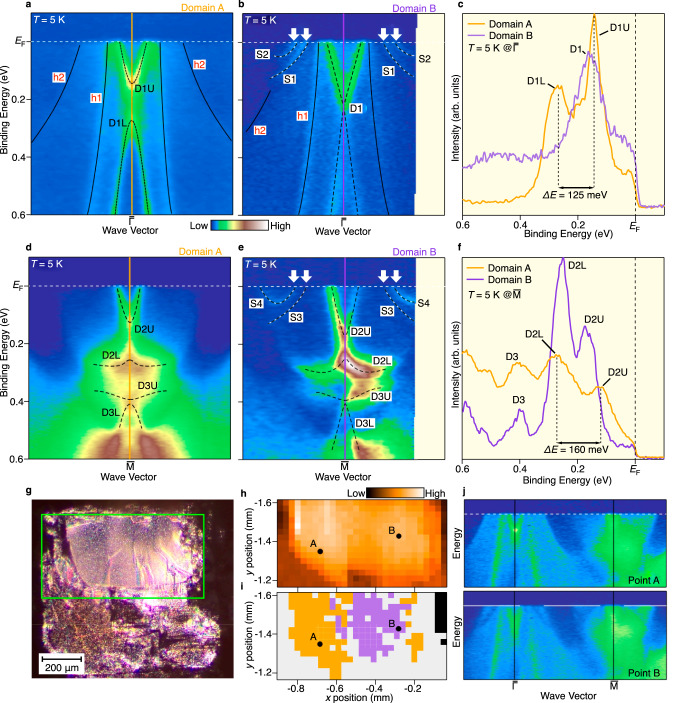
Domain-dependent band structure of NdBi. **a**, **b** ARPES intensity around the $\bar{\Gamma }$ point for domains A and B, respectively. **c** Comparison of EDC at the $\bar{\Gamma }$ point at *T* = 5 K between two domains (orange for domain A and purple for domain B). **d**–**f** Same as (**a**–**c**), but at the $\bar{{{{{{\rm{M}}}}}}}$ point. **g** Optical microscope image for a cleaved surface of NdBi where domain-selective micro-ARPES measurements were performed. **h** Spatial mapping of the ARPES intensity integrated the (*E*, **k**) area around the $\bar{\Gamma }$ point, measured for the area enclosed by the green rectangle in (**g**). **i** Distribution of domains A (orange) and B (purple) estimated from the spatially resolved ARPES-derived band structure. Gray and black colors represent indistinguishable and very-weak-intensity areas, respectively. **j** Representative ARPES intensity in the AF state obtained at specific real-space points A and B in (**h**, **i**). Note that the statistics in (**j**) are poorer because many lateral positions with a mesh of 34 × 18 (in total ~600 data points) had to be covered for the spatial mapping.

Since NdBi crystal is expected to inherently contain multiple AF domains at the surface without magnetic field owing to the cubic structure (Fig. 1a), we surveyed the band structure in the AF phase by scanning a micro-focused beam on the surface and found that there exists another domain (called domain B) that exhibits a spectral feature markedly different from that discussed above (called domain A). As shown in Fig. 3a, on domain A, the bulk h1 and h2 bands outside the D1 band smoothly disperse toward *E*_F_ without anomalies, resembling the band dispersion of the PM phase (Fig. 1f). On the other hand, the energy bands of domain B look significantly reconstructed (Fig. 3b). While the h1 band shows a similar dispersion for two domains, the broad feature originating from the h2 band seen in domain A (Fig. 3a) turns into a couple of sharp features (S1 and S2; white arrows) crossing *E*_F_ with a shallower dispersion. The S1 and S2 bands were assigned to the magnetically split Fermi-arc SS^42^.

The definitive domain-dependent nature also shows up in the Dirac-cone SS, D1. Although domain A hosts a Dirac gap of 125 meV (Figs. 3a and 2d), an X-shaped band with no signature for a Dirac gap is observed in domain B (Fig. 3b). A critical difference is also visualized by a comparison of the EDC at *T* = 5 K in Fig. 3c which signifies a double peak for domain A in contrast to a single peak for domain B. This suggests symmetry protection of the Dirac-cone SS (D1) in domain B despite the time-reversal-symmetry breaking. In contrast to the D1 band, the D2 band at the $\bar{{{{{{\rm{M}}}}}}}$ point gapped out by 160 meV for domain A (Fig. 3d) still shows the band separation (D2U and D2L) by 80 meV for domain B (Fig. 3e). Persistence of the gap of the D2 band is also visualized by the EDCs (Fig. 3f) which signify the double-peaked structure (D2U and D2L) for both domains (note that the D3 peak at *E*_B_ ~ 0.4 eV is sharper for domain B than domain A, indicative of a change in the gap magnitude, although its quantitative estimation is difficult). According to the *Z*_2_ classification, when the number of Dirac-cone SS is odd on a given surface, the Dirac cone is topologically protected, whereas it is not protected for the even-number case^8,12,13^. In the present case for domain B, the single Dirac-cone SS (D1) is protected, whereas the other two Dirac-cone SSs (D2 and D3) are not. Thus, the residual gap opening for the D2 band may not violate the Z_2_ topological protection, but indicates its topologically more fragile nature. This point needs to be further examined, as detailed in Supplementary Note 2.

We systematically scanned micro-focused VUV photons on the cleaved surface (Fig. 3g), and probed the local band structure as a function of real-space position (*x*, *y*) in the AF phase, as highlighted by the mapping of photoelectron intensity around the $\bar{\Gamma }$ point (Fig. 3h). As a result, the ARPES spectra in the AF phase were categorized into either domain A, domain B, or other indistinguishable region, as indicated by different colorings in Fig. 3i. The distinction of domains was made by looking at the local band dispersion together with specifying the aforementioned anomalies in the h2 and D1 bands, as exemplified in Fig. 3j. We found that the ARPES spectrum in the PM phase shows no meaningful (*x*, *y*) dependence, indicative of the disappearance of such domains, confirming the AF origin of domains A and B. Such two types of domains have not been resolved in the previous ARPES study of NdBi^42^, while some micro-ARPES studies have been already applied to other rare-earth pnictides such as CeSb^52,53^. The existence of multiple AF domains in NdBi was also confirmed by our polarizing microscope measurements, as detailed in Supplementary Note 5 and Supplementary Fig. 6.

### *S*-symmetry protection of the Dirac-cone SS

**Fig. 4:**
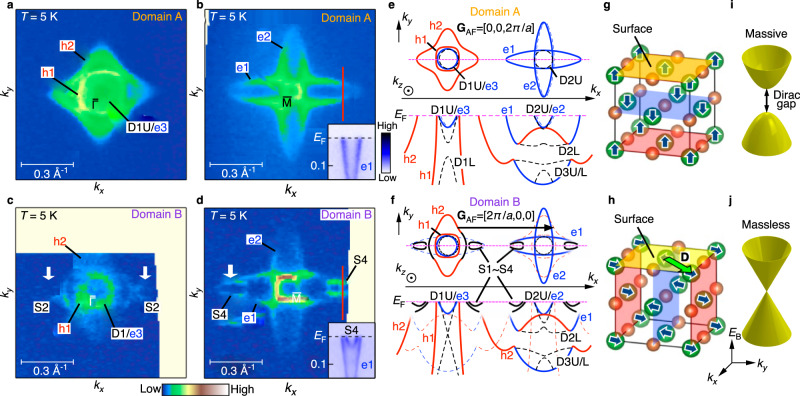
Schematics of the relationship between two AF domains and electronic states. **a**, **b** ARPES intensity around the $\bar{\Gamma }$ and $\bar{{{{{{\rm{M}}}}}}}$ points, respectively, for domain A. Inset to **b** shows the near-*E*_F_ ARPES intensity along a **k** cut shown by the red line. **c**, **d** Same as (**a**, **b**) but for domain B. **e**, **f** Schematics of the influence of AF structure to the folding of FS and band dispersion for domains A and B, respectively. **G**_AF_ is a reciprocal lattice vector of magnetic BZ for each domain. Red, blue, and black curves represent energy bands for bulk hole, bulk electron, and surface bands, respectively. **g**, **h** AF structure for domains A and B, respectively. Translation vector **D** inverting the spin direction is indicated by the green arrow in (**h**). **i**, **j** Corresponding schematic band dispersion of Dirac-cone bands for domains A and B, respectively.

To obtain further insights into the origin of intriguing domain-dependent electronic structure, we directly compare the FS topology between domains A and B as shown in Fig. 4a–d. By a side-by-side comparison of the FS around the $\bar{{{{{{\rm{M}}}}}}}$ point (Fig. 4b, d), one can recognize that there exist small pockets at both corners of the horizontally elongated electron pocket, e1, only for domain B (white arrows). In fact, the ARPES intensity along a **k** cut crossing this pocket (red lines in Fig. 4b, d) signifies an additional shallow electron band that likely arises from the SS (S4)^42,43^ besides the e1 band (inset to Fig. 4d) whereas it is completely absent for domain A (inset to Fig. 4b). Around the $\bar{\Gamma }$ point, while domain A shows a normal FS image (Fig. 4a) similar to the case of PM phase (Fig. 1e), domain B obviously has an anomaly at both corners of the h2 pocket (Fig. 4c); a small pocket (S2) that resembles the pocket of the $\bar{{{{{{\rm{M}}}}}}}$-centered FS (Fig. 4d) seems to appear.

The observed critical differences in the FS topology between the two domains are explained by taking into account the existence of two types of AF domains at the same surface and the relevant band reconstruction, as highlighted in Fig. 4e–h. Our data for domain A are consistent with the magnetic structure in which the AF stacking occurs along the out-of-plane direction and the magnetic moment of Nd ions at the topmost surface layer aligns ferromagnetically (Fig. 4g). Since the observed bulk-band structure is significantly broadened along *k*_*z*_ already in the PM phase, it does not show an effective change even when entering into the AF phase because the band folding occurs along the *k*_*z*_ direction (Fig. 4e). The C_4_ symmetric pattern in Fig. 4a also supports the AF-ordering along *k*_*z*_. On the other hand, our data for domain B are consistent with the magnetic structure in which the AF stacking occurs along the in-plane direction (e.g., along the *x*-axis), corresponding to the antiparallel configuration of the magnetic moment at the topmost layer (Fig. 4h). In this case, the band folding occurs with respect to the magnetic BZ boundary at halfway between the $\bar{\Gamma }$ and $\bar{{{{{{\rm{M}}}}}}}$ points (*k*_*x*_ = *π*/*a*; Fig. 4f). Consequently, the e1 pocket around $\bar{{{{{{\rm{M}}}}}}}$ is folded to $\bar{\Gamma }$, and the h1 and h2 pockets around $\bar{\Gamma }$ to $\bar{{{{{{\rm{M}}}}}}}$ (note that the intensity of the folded bulk bands is weak). Because the magnetic BZ has the C_2_ symmetry along the *k*_*x*_ axis, small pockets associated with the AF-induced SS (S1–S4) appear only at the horizontal side of the original bulk pocket, reflecting the magnetic BZ boundary (see Supplementary Note 6 for details). We have confirmed that the C_2_ symmetric electronic structure is not associated with the matrix-element effect of photoelectron intensity, as detailed in Supplementary Note 7. Our argument is further corroborated by the domain-selective ARPES measurements on a cousin material NdSb^54,55^ which signified the existence of all three types of AF domains (out-of-plane, in-plane horizontal/vertical) together with the non-topological (non *Z*_2_) nature of the S1–S4 pockets. This conclusion is different from that of the recent study which suggests the C_4_ symmetric FS^42^, probably because of the better spatial resolution of the present study.

The configuration of the AF order plays a crucial role in the massive vs massless characteristics of the Dirac-cone SS. When the translation vector (**D**) lies on the surface (domain B; Fig. 4h), the combined symmetry *S* = *ΘT*_D_ is expected to be preserved and the Dirac cone is protected^8,12,13^. As we demonstrated in Fig. 3a–f, although the time-reversal symmetry (*Θ*) is broken in the AF phase, the D1 band for domain B is still protected by the *S* symmetry and maintains the massless character (Fig. 4j), in contrast to the massive character for domain A whose surface breaks the *S* symmetry (Fig. 4i). Such distinction, which owes to the high spatial resolution of micro-focused ARPES, together with our first-principles band-structure calculations that signify the domain-dependent Dirac gap (for details, see Supplementary Note 2), firmly verifies the *S*-symmetry protection of the Dirac cone, and thus validates the long-awaited AF TI phase proposed by theory^8,9^.

Topological protection by the *S* symmetry suggests a high tunability of the Dirac-cone SS via controlling the AF domain and the surface index^8,15,19^. Such tunability would be useful for realizing exotic topological matters such as 3D axion insulators and higher-order TI. For example, when we cut a NdBi crystal so as to make all facets have a domain-A-type configuration where the **D** vector contains a finite out-of-plane component, an axion insulator with a negative band gap may be realized^8,15,19^. Further, on a certain crystal hinge between these facets, a 1D chiral hinge state may emerge within the surface Dirac gap—a signature of higher-order TI^11,15,19^. At the step edge of the ferromagnetic layer, 1D chiral edge mode is proposed to appear^8,20,25^. Thus, besides the significance of the discovery of AF TI protected by the *S* symmetry, NdBi also serves as a useful platform to realize exotic quantum states.

## Methods

### Sample fabrication

Single crystals of NdBi were grown by the flux method using indium flux. The raw materials were mixed in a molar ratio of Nd:Bi:In = 1:1:20 and placed in an alumina crucible. The crucible was sealed in an evacuated Quartz tube filled with Ar gas of 50 mbar. The ampoule was heated to 1100 °C, kept for 10 h, and then cooled to 700 °C in 160 h. The excessive indium was removed in a centrifuge. Obtained crystals were characterized by X-ray diffraction measurements.

### ARPES measurements

SX-ARPES measurements were performed with an Omicron-Scienta SES2002 electron analyzer with energy-tunable synchrotron light at BL2 in Photon Factory (PF), KEK. We used linearly polarized light (horizontal polarization) of 515–601 eV. VUV-ARPES measurements were performed with micro-focused VUV synchrotron light at BL28 in PF^56^, BL5U in UVSOR, and I05 in Diamond Light Source. We used linearly or circularly polarized light of 44–75 eV. The energy resolution for the SX- and VUV-ARPES measurements was set to be 150 and 10–20 meV, respectively. Samples were cleaved in situ along the (001) plane of the cubic crystal in an ultrahigh vacuum of 1 × 10^−10 ^Torr. Prior to the ARPES measurement, the crystal orientation was determined by the X-ray Laue backscattering measurement which signifies clear four-fold symmetric diffraction spots consistent with the (001) cleaved plane. The Fermi level (*E*_F_) of samples was referenced to that of a gold film electrically in contact with the sample holder.

### Calculations

First-principles band-structure calculations were carried out by using a projector augmented wave method implemented in Vienna Abinitio Simulation Package (VASP) code^57^. To calculate the band structure for the PM phase, the modified Becke–Johnson (mBJ) potential^58^ which is known to properly reproduce the band gap in RX_p_^59^, was used for the exchange-correlation functional. The total energy was calculated self-consistently with the tetrahedron sampling of 8 × 8 × 1 *k*-point mesh taking into account SOC. The surface states were obtained with the surface Green’s function method implemented in WannierTools code^60^ after the maximally localized Wannier functions for Bi-*s*, Bi-*p*, and Nd-*d* orbital states were obtained by using Wannier90 code^61^. For the AF phase, we have carried out slab calculations with 12 atomic-layer slabs by taking into account the actual type-I AF structure (Supplementary Figs. 2 and 3). To properly take into account the magnetic moment of Nd ions, we included the strong correlation effect of Nd 4*f* electrons by using GGA+*U* calculation potential^62^ instead of mBJ potential which has a nonconvergence problem in the slab calculations as in the previous study^57^. We have carried out the unfolding of bands for the superstructure in the AF phase for domain B by using a method proposed in the previous literature^63^.

### Polarizing microscopy

Polarizing microscopy measurements were performed by using a home-built UHV microscope system at Tohoku University. We have used a 100 W halogen lamp (U-LH100L-3, Olympus) to obtain bright reflectance images. Polarizing images were obtained in the crossed Nicols configuration with the optical principal axes along [110] axis. Samples were cleaved in situ along the (001) plane of cubic crystal in a UHV of 1 × 10^−10 ^Torr. The sample was cooled by liquid helium cryostat and the temperature was controlled in the range of 5–30 K.

### Supplementary information

Supplementary Information

Peer Review File

## Data Availability

The data that support the findings of this study are available within the main text and Supplementary Information. Any other relevant data are available from the corresponding authors upon request.

## References

[1] Qi X-L, Hughes TL, Zhang S-C (2008). Topological field theory of time-reversal invariant insulators. Phys. Rev. B.

[2] Yu R (2010). Quantized anomalous Hall effect in magnetic topological insulators. Science.

[3] Nomura K, Nagaosa N (2011). Surface-quantized anomalous Hall current and the magnetoelectric effect in magnetically disordered topological insulators. Phys. Rev. Lett..

[4] Tokura Y, Yasuda K, Tsukazaki A (2019). Magnetic topological insulators. Nat. Rev. Phys..

[5] Chang C-Z (2013). Experimental observation of the quantum anomalous Hall effect in a magnetic topological insulator. Science.

[6] Checkelsky JG (2014). Trajectory of the anomalous Hall effect towards the quantized state in a ferromagnetic topological insulator. Nat. Phys..

[7] Kou X (2014). Scale-invariant quantum anomalous Hall effect in magnetic topological insulators beyond the two-dimensional limit. Phys. Rev. Lett..

[8] Mong RSK, Essin AM, Moore JE (2010). Antiferromagnetic topological insulators. Phys. Rev. B.

[9] Li R, Wang J, Qi X-L, Zhang SC (2010). Dynamical axion field in topological magnetic insulators. Nat. Phys..

[10] Varnava N, Wilson JH, Pixley JH, Vanderbilt D (2021). Controllable quantum point junction on the surface of an antiferromagnetic topological insulator. Nat. Commun..

[11] Peng Y, Xu Y (2019). Proximity-induced Majorana hinge modes in antiferromagnetic topological insulators. Phys. Rev. B.

[12] Fang C, Gilbert MJ, Bernevig BA (2013). Topological insulators with commensurate antiferromagnetism. Phys. Rev. B.

[13] Zhang R-X, Liu C-X (2015). Topological magnetic crystalline insulators and corepresentation theory. Phys. Rev. B.

[14] Otrokov MM (2019). Prediction and observation of an antiferromagnetic topological insulator. Nature.

[15] Xu Y, Song Z, Wang Z, Weng H, Dai X (2019). Higher-order topology of the axion insulator EuIn_2_As_2_. Phys. Rev. Lett..

[16] Watanabe H, Po HC, Vishwanath A (2018). Structure and topology of band structures in the 1651 magnetic space groups. Sci. Adv..

[17] Xu Y (2020). High-throughput calculations of magnetic topological materials. Nature.

[18] Turner AM, Zhang Y, Mong RSK, Vishwanath A (2012). Quantized response and topology of magnetic insulators with inversion symmetry. Phys. Rev. B.

[19] Varnava N, Vanderbilt D (2018). Surfaces of axion insulators. Phys. Rev. B.

[20] Essin AM, Moore JE, Vanderbilt D (2009). Magnetoelectric polarizability and axion electrodynamics in crystalline insulators. Phys. Rev. Lett..

[21] Marsh DJE, Fong KC, Lentz EW, Šmejkal L, Ali MN (2019). Proposal to detect dark matter using axionic topological antiferromagnets. Phys. Rev. Lett..

[22] Šmejkal L, Mokrousov Y, Yan B, MacDonald AH (2018). Topological antiferromagnetic spintronics. Nat. Phys..

[23] Sekine A, Nomura K (2021). Axion electrodynamics in topological materials. J. Appl. Phys..

[24] Bernevig BA, Felser C, Beidenkopf H (2022). Progress and prospects in magnetic topological materials. Nature.

[25] Zhang D (2019). Topological axion states in the magnetic insulator MnBi_2_Te_4_ with the quantized magnetoelectric effect. Phys. Rev. Lett..

[26] Hua G (2018). Dirac semimetal in type-IV magnetic space groups. Phys. Rev. B.

[27] Soh J-R (2019). Ideal Weyl semimetal induced by magnetic exchange. Phys. Rev. B.

[28] Deng Y (2020). Quantum anomalous Hall effect in intrinsic magnetic topological insulator MnBi_2_Te_4_. Science.

[29] Zeugner A (2019). Chemical aspects of the candidate antiferromagnetic topological insulator MnBi_2_Te_4_. Chem. Mater..

[30] Shikin AM (2020). Nature of the Dirac gap modulation and surface magnetic interaction in axion antiferromagnetic topological insulator MnBi_2_Te_4_. Sci. Rep..

[31] Vidal RC (2019). Surface states and Rashba-type spin polarization in antiferromagnetic MnBi_2_Te_4_ (0001). Phys. Rev. B.

[32] Lee SH (2019). Spin scattering and noncollinear spin structure-induced intrinsic anomalous Hall effect in antiferromagnetic topological insulator MnBi_2_Te_4_. Phys. Rev. Res..

[33] Lu R (2021). Half-magnetic topological insulator with magnetization-induced Dirac gap at a selected surface. Phys. Rev. X.

[34] Chen B (2019). Intrinsic magnetic topological insulator phases in the Sb doped MnBi_2_Te_4_ bulks and thin flakes. Nat. Commun..

[35] Li H (2019). Dirac surface states in intrinsic magnetic topological insulators EuSn_2_As_2_ and MnBi_2*n*_Te_3*n*+1_. Phys. Rev. X.

[36] Hao Y-J (2019). Gapless surface Dirac cone in antiferromagnetic topological insulator MnBi_2_Te_4_. Phys. Rev. X.

[37] Chen YJ (2019). Topological electronic structure and its temperature evolution in antiferromagnetic topological insulator MnBi_2_Te_4_. Phys. Rev. X.

[38] Swatek P (2020). Gapless Dirac surface states in the antiferromagnetic topological insulator MnBi_2_Te_4_. Phys. Rev. B.

[39] Liang A (2022). Approaching a minimal topological electronic structure in antiferromagnetic topological insulator MnBi_2_Te_4_ via surface modification. Nano Lett..

[40] Ma X-M (2020). Hybridization-induced gapped and gapless states on the surface of magnetic topological insulators. Phys. Rev. B.

[41] Schobinger-Papamantellos P, Fischer P, Vogt O, Kaldis E (1973). Magnetic ordering of neodymium monopnictides determined by neutron diffraction. J. Phys. C Solid State Phys..

[42] Schrunk B (2022). Emergence of Fermi arcs due to magnetic splitting in an antiferromagnet. Nature.

[43] Li P (2023). Origin of the exotic electronic states in antiferromagnetic NdSb. npj Quantum Mater..

[44] Hasegawa A (1985). Fermi surface of LaSb and LaBi. J. Phys. Soc. Jpn..

[45] Kumigashira H (1997). Paramagnetic-to-antiferroparamagnetic phase transition of CeSb studied by high-resolution angle-resolved photoemission. Phys. Rev. B.

[46] Oinuma H (2017). Three-dimensional band structure of LaSb and CeSb: Absence of band inversion. Phys. Rev. B.

[47] Niu XH (2016). Presence of exotic electronic surface states in LaBi and LaSb. Phys. Rev. B.

[48] Lou R (2017). Evidence of topological insulator state in the semimetal LaBi. Phys. Rev. B.

[49] Nayak J (2017). Multiple Dirac cones at the surface of the topological metal LaBi. Nat. Commun..

[50] Oinuma H (2019). Unusual change in the Dirac-cone energy band upon a two-step magnetic transition in CeBi. Phys. Rev. B.

[51] Kuroda K (2018). Experimental determination of the topological phase diagram in cerium monopnictides. Phys. Rev. Lett..

[52] Kuroda K (2020). Devil’s staircase transition of the electronic structures in CeSb. Nat. Commun..

[53] Arai Y (2022). Multipole polaron in the devil’s staircase of CeSb. Nat. Mater..

[54] Honma A (2023). Unusual surface states associated with *PT*-symmetry breaking and antiferromagnetic band folding in NdSb. Phys. Rev. B.

[55] Kushnirenko Y (2023). Directional effects of antiferromagnetic ordering on the electronic structure in NdSb. Phys. Rev. B.

[56] Kitamura M (2022). Development of a versatile micro-focused angle-resolved photoemission spectroscopy system with Kirkpatrick–Baez mirror optics. Rev. Sci. Instrum..

[57] Kresse G, Furthmüller J (1996). Efficient iterative schemes for ab initio total-energy calculations using a plane-wave basis set. Phys. Rev. B.

[58] Becke AD, Johnson ER (2006). A simple effective potential for exchange. J. Chem. Phys..

[59] Li P (2018). Tunable electronic structure and surface states in rare-earth monobismuthides with partially filled f shell. Phys. Rev. B.

[60] Wu Q, Zhang S, Song H-F, Troyer M, Soluyanov AA (2018). WannierTools: an open-source software package for novel topological materials. Comput. Phys. Commun..

[61] Mostofi AA (2008). wannier90: A tool for obtaining maximally-localised Wannier functions. Comput. Phys. Commun..

[62] Perdew JP, Burke K, Ernzerhof M (1996). Generalized gradient approximation made simple. Phys. Rev. Lett..

[63] Dirnberger D, Kresse G, Franchini C, Reticcioli M (2021). Electronic state unfolding for plane waves: energy bands, fermi surfaces, and spectral functions. J. Phys. Chem. C.

