# Supplementary material for: Antiferromagnetic topological insulator with selectively gapped Dirac cones
Source: Nat Commun. 2023 Nov 17;14:7396. doi: 10.1038/s41467-023-42782-6 (PMC10656484; doi:10.1038/s41467-023-42782-6)
Supplement: Supplementary file 1 — Supplementary Information [file 41467_2023_42782_MOESM1_ESM.pdf]

SUPPLEMENTARY INFORMATION for

"Antiferromagnetic topological insulator with selectively gapped Dirac cones" by A. Honma et al.

Supplementary Note 1: **Bulk electronic structure and band inversion of NdBi**

To visualize the bulk bands of NdBi in three-dimensional (3D) *k* space, it is useful to use bulk-sensitive soft-X-ray (SX) photons because the longer photoelectron mean-free path relative to that for vacuum ultraviolet (VUV) photons reduces the intrinsic uncertainty of the out-of-plane wave vector k_z through the Heisenberg's uncertainty principle and as a result allows the accurate 3D band mapping. Supplementary Fig. 1a and 1b show ARPES-intensity mapping at E_F as a function of in-plane wave vector measured at $T = 40$ K [paramagnetic (PM) phase] for two representative k_z slices in the bulk Brillouin zone (BZ) (see Fig. 1b of the main text) at $k_z \sim 2\pi/a$ ($h\nu = 601$ eV) and ~ 0 ($h\nu = 515$ eV), respectively. One can identify different intensity distributions between $k_z \sim 0$ and $2\pi/a$, signifying that the ARPES signal actually reflects the bulk Fermi surface. At $k_z \sim 0$ (Supplementary Fig. 1a), we find bright intensity spots centered at the Γ point associated with the bulk inner (h1) and outer (h2) hole pockets, together with a weaker intensity centered at the X point elongated along the ΓX direction attributable to the bulk electron pockets (e1 and e2). The hole and electron pockets originate from the topmost bulk valence bands with the Bi-6*p* orbital character and the lowest bulk conduction band with the Nd-5*d* character, respectively, which are responsible for the semimetallic nature of rare-earth monopnictides[1] as schematically shown in Fig. 1i of the main text. Since the bulk-band inversion at the X point is known to be directly linked to the topological nature[2–9], we show in Supplementary Fig. 1c (left panel) the ARPES intensity and (right panel) the corresponding second derivative intensity along the ΓX cut of bulk 3D BZ measured at $h\nu = 515$ eV. The result signifies a weak Nd 5*d* electron band e1 (dashed blue curve) which crosses E_F midway between the Γ and X points, together with the inner (h1) and outer (h2) Bi-6*p* hole bands around the Γ point. The e1 and h2 bands appear to show a hybridization gap at the intersecting point (white arrow), suggestive of the inverted band structure as in the case of LaBi and CeBi[4,9].

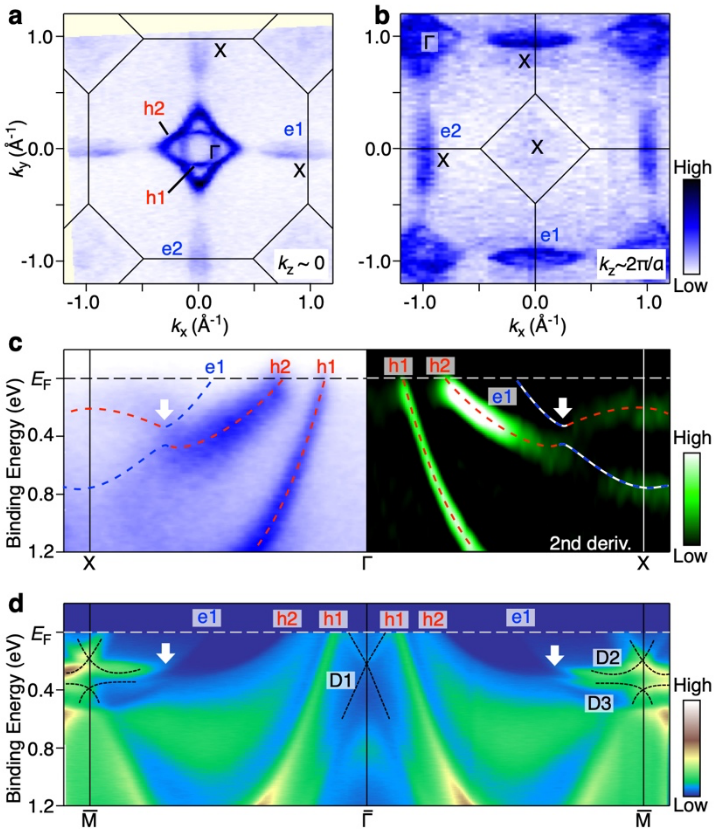

Supplementary Fig. 1 | Bulk band structure and band inversion of NdBi. a, b ARPES-intensity mapping at E_F as a function of in-plane wave vector (k_x and k_y) measured at $T = 40$ K at $k_z \sim 2\pi/a$ ($h\nu = 601$ eV) and ~ 0 ($h\nu = 515$ eV), respectively. **c** ARPES intensity (left) and corresponding second derivative intensity (right) plotted as a function of k_x and binding energy (E_B) along the ΓX cut of bulk BZ measured with SX photons of $h\nu = 515$ eV. Red and blue dashed curves are a guide for the eyes to trace the Bi-6*p* (h1 and h2) and Nd 5*d* (e1) bands, respectively. **d** ARPES intensity along the $\bar{\Gamma}\bar{M}$ cut of surface BZ measured at $T = 35$ K with VUV photons of $h\nu = 60$ eV. Black dashed curves are a guide for the eyes to trace the surface band dispersions.

To signify the correspondence between the bulk band inversion and the Dirac-cone surface state (SS), we have mapped the ARPES intensity with surface-sensitive VUV photons ($h\nu = 60$ eV) along the same ΓX cut (corresponding to the $\bar{\Gamma}\bar{M}$ cut in the surface BZ). Although the intensity of h1 and h2 bands is broadly distributed due to the short photoelectron escape depth and resultant strong k_z broadening[2,9,10], one can clearly identify in Supplementary Fig. 1d the band-inversion-originated anomaly at the intersection of the Nd 5*d* and Bi-6*p* bands (white arrows) which reflects the hybridization

of the e1 and h2 bands. Owing to the surface sensitivity of VUV photons, one can identify a weak Dirac-cone band (D1) around the $\bar{\Gamma}$ point inside the h1 band, as well as two Dirac-cone bands (D2 and D3) around the $\bar{M}$ point. Those bands (D1–D3) are not well resolved in the bulk-sensitive SX data (Supplementary Fig. 1c), consistent with their surface origin. Energy levels of Nd 5*d* and Bi-6*p* bands are inverted at the X point, one of the time-reversal-invariant momentum (TRIM), producing the Dirac-cone SS protected by time-reversal symmetry[2–6,9]. Associated with the difference in the number of bulk-band inversions projected onto the high-symmetry **k** points in the surface BZ, the number of Dirac-cone SS are different between the $\bar{\Gamma}$ and $\bar{M}$ points. Specifically, the D1 band at $\bar{\Gamma}$ is associated with the band inversion at single X point [$\mathbf{k} = (0, 0, 2\pi/a)$] of the bulk BZ, whereas the D2 and D3 bands are with the band inversions at two X points [$\mathbf{k} = (2\pi/a, 0, 0)$ and $(2\pi/a, 0, 2\pi/a)$], as also explained in the main text (Fig. 1b). The odd number of total Dirac-cone SSs at TRIM, $\bar{\Gamma}$ and $\bar{M}$ (which is equivalent to the odd number of band crossings along the $\bar{\Gamma}$ and $\bar{M}$ points) supports the topological insulator nature of NdBi in the paramagnetic phase.

Supplementary Note 2: **Band calculations for the PM and AF phases**

We have carried out first-principles band-structure calculations for NdBi with mBJ potential[11] and GGA potential[12] to support (i) the bulk-band inversion, (ii) the topological nature from the parity analysis, and (iii) the reproduction of D1–D3 SS in the PM phase, and (iv) the reproduction of the Dirac gap in the calculation for the AF phase. Regarding (i), we show in Fig. 1c, d of the main text a direct comparison of the bulk-band dispersion along the ΓX cut between the experiment for the PM phase ($T = 40$ K) and the calculation for the nonmagnetic phase performed with mBJ potential which is known to properly reproduce the band gap in RX_p (ref. 13). Here, Nd-4*f* electrons were treated as core states in the calculation. One can immediately recognize that the overall band structure shows a good agreement between the two. In particular, the experimental hole band topped at the binding energy (E_B) of ~ 1.6 eV and the inner hole band h1 are well reproduced by the calculation. In the experiment, we observe band crossing between the h2 and e1 bands and the resultant spin-orbit gap opening at the intersection. This bulk band inversion is well reproduced in the calculation. Thus, the bulk-band inversion is supported in our band calculation.

Regarding (ii), we calculated the parity eigenvalues ξ for the valence bands to obtain the Z_2 index[14] for NdBi based on the above bulk band calculation. Since band 9 in Fig. 1d is assigned to the conduction band and bands 1–5 form fully occupied closed shells (Nd 5*s*, 5*p* and Bi 6*s*), it is sufficient to consider the topmost three valence bands (bands 6–8) for the parity analysis. Supplementary Table 1 shows ξ at eight time-reversal invariant momenta (TRIM; Γ_i), i.e. Γ, 3X, and 4L. For each TRIM, the δ_i value was obtained by multiplying ξ for bands 6–8, and the Z_2 topological invariants (v_0; v_1, v_2, v_3) were calculated as follows.

$$
\begin{aligned}
(-1)^{v_0} &= \delta(\Gamma)\delta(\mathrm{X})^3\delta(\mathrm{L})^4 && = -1 \\
(-1)^{v_1} &= \delta(\mathrm{X})^2\delta(\mathrm{L})^2 && = 1 \\
(-1)^{v_2} &= \delta(\mathrm{X})^2\delta(\mathrm{L})^2 && = 1 \\
(-1)^{v_3} &= \delta(\mathrm{X})^2\delta(\mathrm{L})^2 && = 1
\end{aligned}
$$

Here, topological invariants v_1, v_2, and v_3 were calculated for the cleaved planes of (100), (010), and (001), respectively, to obtain direct correspondence with the ARPES experiments. The result shows that NdBi is a strong TI in the PM phase with (v_0; v_1, v_2, v_3) = (1; 0, 0, 0), in good agreement with the band inverted character shown in Fig. 1c.

Γ_i / ζ, δ_i	Γ	3X	4L	v_0
$\zeta_8(\Gamma_i)$	+	+	-	
$\zeta_7(\Gamma_i)$	+	-	-	
$\zeta_6(\Gamma_i)$	-	-	-	
δ_i	-	+	-	1

Supplementary Table 1 | Parities of energy bands at 8 TRIMs. Products of parity eigenvalues of the occupied valence-band states (ξ) for bands 6–8, at the time-reversal invariant momenta (TRIMs; Γ_i) of the bulk fcc BZ. δ_i is a product of ξ at 8 TRIMs for all the valence bands. v_0 = 1 indicates the nontrivial Z_2 topology of NdBi in the PM phase.

Regarding (iii), we have carried out tight-binding calculations for 100 atomic layers in the PM phase of NdBi, and calculated the surface projected spectral weight along high symmetry lines of the surface BZ as shown in Fig. 1h of the main text (note that the Nd-4*f* orbitals were assumed to be the core states as in the case of the bulk-band calculations because this assumption was necessary for constructing Wannier functions by fitting the

calculated DFT bands to correctly simulate the surface-projected spectral weight). The result signifies the Dirac-cone SS, D1, at the $\bar{\Gamma}$ point, together with the double Dirac-cone SSs, D2 and D3, at the $\bar{M}$ point, consistent with the ARPES results for the PM phase.

Regarding (iv), we have carried out slab calculations in the AF phase with 12 atomic-layer slabs by taking into account the actual type-I AF structure (Supplementary Fig. 2a). To properly take into account the magnetic moment of Nd ions, we included the strong correlation effect of Nd-4*f* electrons by using GGA+*U* potential instead of mBJ potential which has a nonconvergence problem in the slab calculations as in the previous study[13]. In GGA+*U* slab calculations, the Nd-4*f* bands also appear in the calculation at the binding energy of 5–6 eV (out of the energy range of Supplementary Fig. 2). Since this treatment made it difficult to calculate the surface-projected spectral weight because of the difficulty in constructing the proper Wannier functions by fitting the calculated DFT bands due to the presence of 4*f* states, we show in Supplementary Fig. 2b the original band dispersion for the 12 atomic-layer slab instead of the surface-projected weight. Although obtaining a one-to-one correspondence in the calculated band structure with the PM state in Fig. 1h is difficult, for the top surface corresponding to domain A in the

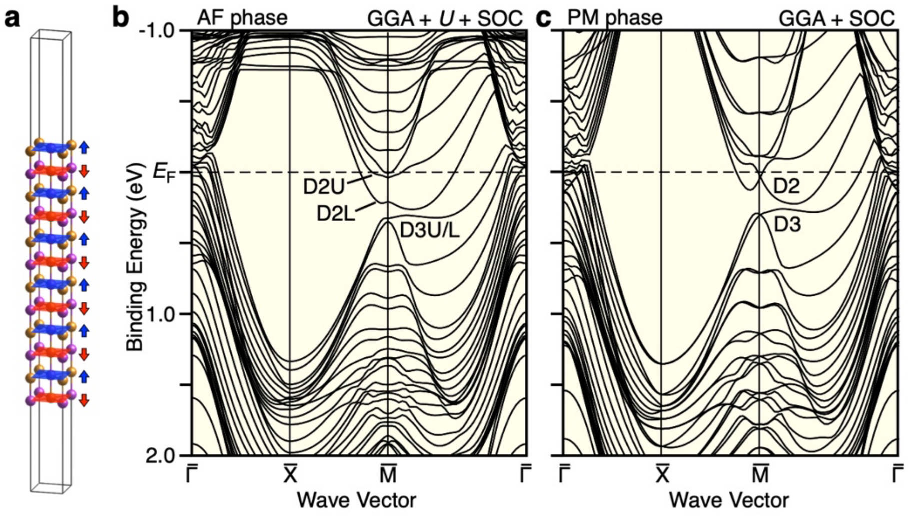

Supplementary Fig. 2 | Calculated band structure in the AF phase. a 12 atomic layer slab structure with type-I AF configuration adopted for the calculations to simulate the band structure for domain A. **b**, **c** Calculated band structure in the AF and PM phases along high-symmetry lines in the surface BZ. Possible D2 and D3 bands are also indicated.

ARPES data, one can see a signature of the D2 and D3 bands near E_F. Intriguingly, an energy gap opens at the $\bar{M}$ point for both the D2 and D3 bands. This gap is confirmed to be of AF origin because the calculation for the PM phase obtained for the same 12 atomic-layer slab in Supplementary Fig. 2c clearly shows the gapless behavior due to the protection by the time-reversal symmetry. Also, the gap for the D2 band shown in Supplementary Fig. 2b is significantly enhanced relative to that of the D3 band. These key features are nicely reproduced in the ARPES experiment (Fig. 2h and 2i in the main text). It is noted here that the validation of the Dirac gap for the D1 band was difficult because of the overlap with the bulk bands, as shown in Fig. 1h. We have carried out slab calculations for the AF phase also for domain B (Supplementary Fig. 3a) to further validate the concept of *S*-symmetry protection. The band structure unfolded to the original BZ shown in Supplementary Fig. 3b signifies that the Dirac gap for the D2 and D3 bands is absent, distinct from domain A (Supplementary Fig. 2). This theoretically demonstrates the selectively gapped Dirac-cone state and supports the validity of *S*-symmetry protection. Such an intriguing surface-dependent Dirac gap is overall consistent with the

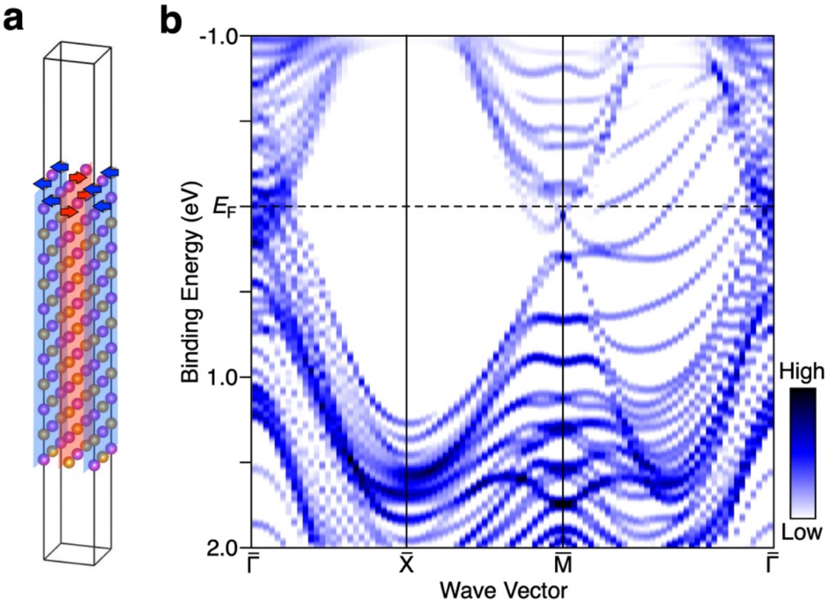

Supplementary Fig. 3 | Calculated band structure in the AF phase for domain B. a 12 atomic layer slab model with type-I AF configuration for domain B. $\sqrt{2}\times\sqrt{2}$ supercell is adopted to simulate the transverse AF order. **b** Intensity of spectra corresponds to the amplitude of projection to the original cell eigenstate.

ARPES observation shown in Fig. 3a–f, whereas the origin of the residual gap for the D2 band in domain B (Fig. 3f) remains as an open question. The deviation from the theory may invoke exotic mechanisms beyond the present DFT calculation, such as modulation of the surface magnetic structure, spin fluctuations, and strong many-body effects. We leave this issue as a challenge in the future study.

All these arguments regarding (i) the bulk-band inversion commonly identified in the experiment and calculation, (ii) the strong TI [$(\nu_0; \nu_1, \nu_2, \nu_3) = (1; 0, 0, 0)$] nature in the PM phase from the parity analysis, (iii) the reproduction of D1–D3 SS by the slab calculations, and (iv) the Dirac-gap opening for the calculated D2 and D3 SS in the AF state for domain A, support and strengthen our main claim on the AF TI nature of NdBi.

Supplementary Note 3: ***k_z*-independent energy dispersion of the Dirac-cone SS**

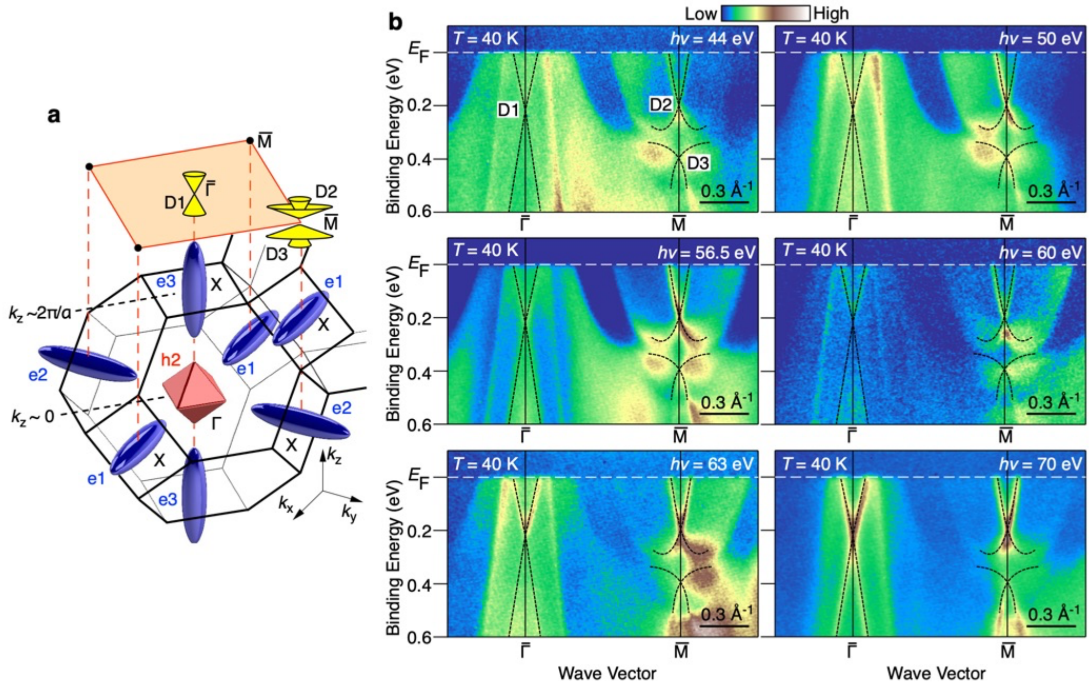

Supplementary Fig. 4 | *k_z*-independent energy dispersion of the Dirac-cone SS in NdBi. a Schematic FS and bulk fcc BZ of NdBi, together with the surface BZ projected onto the (001) plane (orange rectangle) and Dirac-cone SS at the $\bar{\Gamma}$ and $\bar{\mathrm{M}}$ points (same as Fig. 2c in the main text). **b** *hν*-dependence of the ARPES intensity along the $\bar{\Gamma}\bar{\mathrm{M}}$ cut of surface BZ, measured in the PM phase (T = 40 K) at *hν* = 44–70 eV. Dashed curves are a guide for the eyes that traces the experimental band dispersion of the D1–D3 bands obtained at *hν* = 75 eV (same as Fig. 2c, h in the main text).

We have carried out $h\nu$-dependent ARPES measurements to experimentally demonstrate the surface nature of the Dirac-cone state by measuring the k_z dispersion with several photon energies ($h\nu$'s). Since the intensity of D1–D3 bands was found to be strongly suppressed for the bulk-sensitive soft X-ray (SX) photons (see Supplementary Fig. 1), we measured the detailed $h\nu$ dependence with the surface-sensitive vacuum ultraviolet (VUV) photons at $h\nu$ = 44, 50, 56.5, 60, 63, 70 eV, besides $h\nu$ = 75 eV (Fig. 2c of the main text). This $h\nu$ range fully covers the ΓX length of the bulk Brillouin zone (k_z = 0 to $2\pi/a$) with a reasonably small k_z step. We found from the ARPES intensity obtained in the PM phase at T = 40 K shown in Supplementary Fig. 4 that the energy position of the D1–D3 bands estimated from the ARPES data at $h\nu$ = 75 eV (same as dashed curves in Fig. 2c and 2h of the main text) overlaps with the intensity of the D1–D3 bands irrespective of $h\nu$'s, despite the strong modulation of their intensities associated with the matrix-element effect. This result strongly suggests the surface origin of the D1–D3 bands.

Supplementary Note 4: **AF origin of the Dirac gap for the D1 band**

We have carried out detailed temperature-dependent ARPES measurements for the D1 band to clarify whether the energy splitting in the AF phase is indeed associated with the AF transition. As shown in Supplementary Fig. 5, one can recognize a clear energy splitting of the upper Dirac-cone (D1U) and lower Dirac-cone (D1L) bands at T = 15 K (Supplementary Fig. 5a1). On increasing temperature, these bands gradually broaden and approach each other. The splitting appears to persist at least up to T = 21 K (Supplementary Fig. 5a7) and becomes invisible at 24 K (= T_N) and 27 K. As shown by the ARPES intensity at the $\bar{\Gamma}$ point plotted against temperature in Supplementary Fig. 5b, the D1 band starts to split into the D1L and D1U bands just at T_N. These results strongly support the AF origin of the Dirac gap.

It is noted here that the energy position of the DP in the PM phase (0.21 eV) in Fig 2c of the main text (as represented by the ARPES data in Supplementary Fig. 4a11) estimated by the linear extrapolation of MDC peak positions slightly deviates from the peak position of the EDC at the $\bar{\Gamma}$ point in Fig. 2e (0.18 eV). We think that this difference is associated with the local deviation of the Dirac-band dispersion from the linear behavior around the DP. The upper Dirac-cone band is rounded around the Dirac point and connected to a highly dispersive lower Dirac-cone band with weaker intensity at the

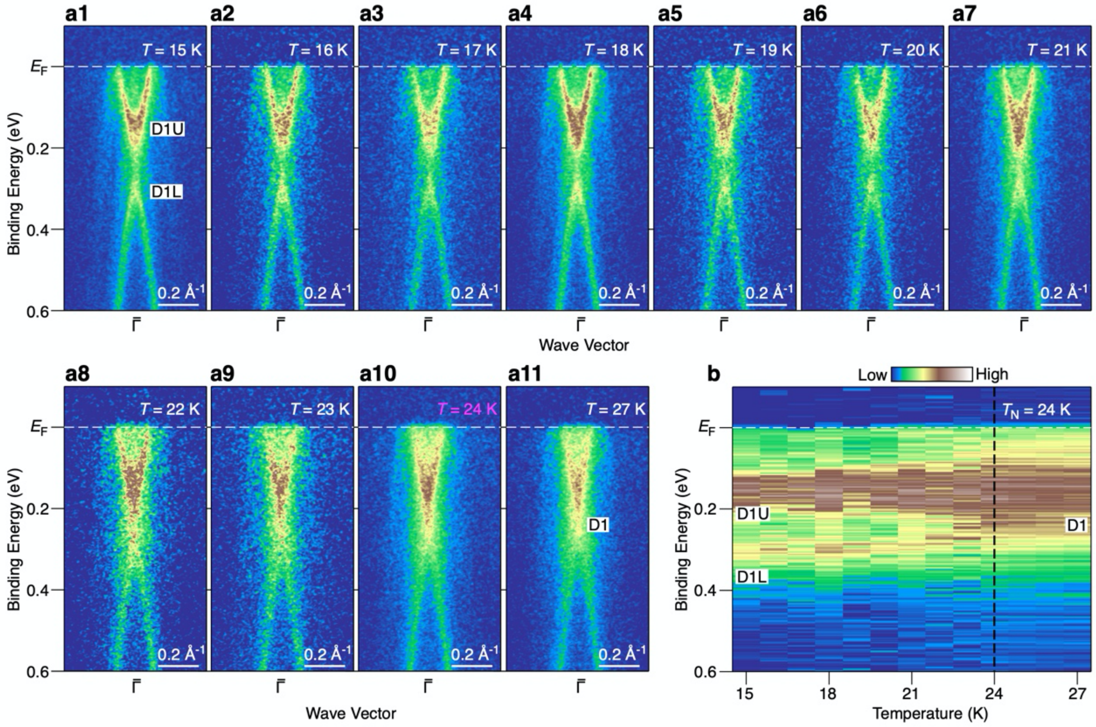

Supplementary Fig. 5 | Temperature dependence of the D1-band dispersion across *T*N. a1–a11 ARPES intensity along the $\overline{\Gamma}\overline{M}$ cut for the D1 band measured at representative temperatures (T = 15–27 K) across the Néel temperature T_N (= 24 K) measured at $h\nu$ = 75 eV. X-shaped gapless Dirac-cone band (D1) in the PM phase turns into the gapped Dirac-cone band (D1U and D1L) in the AF phase. **b** Temperature dependence of the ARPES intensity at the $\overline{\Gamma}$ point which signifies the energy splitting of the D1 band in the AF phase.

$\overline{\Gamma}$ point. Indeed, such behavior was recognized in previous ARPES and DFT-calculation studies in other rare-earth monopnictides[2–5]. Another mechanism to cause a deviation of the DP and EDC-peak energies may be a complication of the overall spectral lineshape (in particular EDCs) due to the strong surface-bulk interaction for the D1 band as supported by our first-principles band calculations.

Here we briefly discuss the reason why the observed gap (125 meV) in the AF state is so large. The gap size in NdBi is indeed much larger than those in other AF TI candidates such as $MnBi_2Te_4$ (MBT) which shows the AF-induced gap of at most 85 meV[15–17] (note that the reported gap size significantly varies depending on the group). While the exact reason for the observed large gap is unclear at the moment, we speculate

that the Dirac gap size is linked to the density and magnetic moment of magnetic ions (Nd/Mn). Specifically, the magnetic moment for Nd^{3+} ion (3.1 μ_B) is ~70 % of that of Mn^{2+} ion (4.6 μ_B), while the magnetic ion density in NdBi (Nd:Bi = 1:1) is 350 % of that of MBT (Mn:Bi_2Te_4 = 1:6). In total, the effective exchange field for the surface Dirac electrons is expected to be much larger in NdBi than in MBT, likely producing a larger gap in NdBi. However, the exchange constants estimated from the neutron diffraction experiments are J_1 = -0.008 meV and J_2 = 0.016 meV for the nearest neighbor and next nearest neighbor, respectively[18], far smaller than the observed Dirac gap. Thus, although a qualitative argument on the larger Dirac gap may be possible, it is hard to quantitatively explain the magnitude of the Dirac gap in NdBi.

Supplementary Note 5: **Identification of AF domains by polarizing microscopy**

We have characterized the AF domain structure of NdBi to substantiate our main claim regarding the AF-domain-dependent Dirac-cone feature by using a low-temperature UHV polarizing microscope system as shown in Supplementary Fig. 6a, b. This system is based on the birefringence of the sample associated with the coupling of optical properties and electronic states, and detects the change in the light polarization between incoming and outgoing reflected photons (Supplementary Fig. 6c), as already applied to other rare-earth monopnictides to detect AF domains[19]. In this system, white light emitted from a halogen lamp is horizontally polarized, and irradiated on the sample. Then, the reflected light passes the vertical polarizer and is detected by a CCD camera (crossed Nicols configuration). Depending on the direction of the Nd-4*f* magnetic moment with respect to the polarization vector of the incident light, three types of AF domains can be distinguished in this system[18]. Supplementary Fig. 6g shows a microscope image subtracted between the PM (Supplementary Fig. 6e) and the AF phases (Supplementary Fig. 6f). One can see domain A with the out-of-plane magnetic moment as a white colored region (i.e. no birefringence) and domain B with the in-plane magnetic moment as a red/blue colored region. Importantly, we found that the spot size of the microbeam shown by the black circle is much smaller than the typical AF domain size. This result supports that the spatially dependent band structure observed by ARPES is related to the difference in the AF domain.

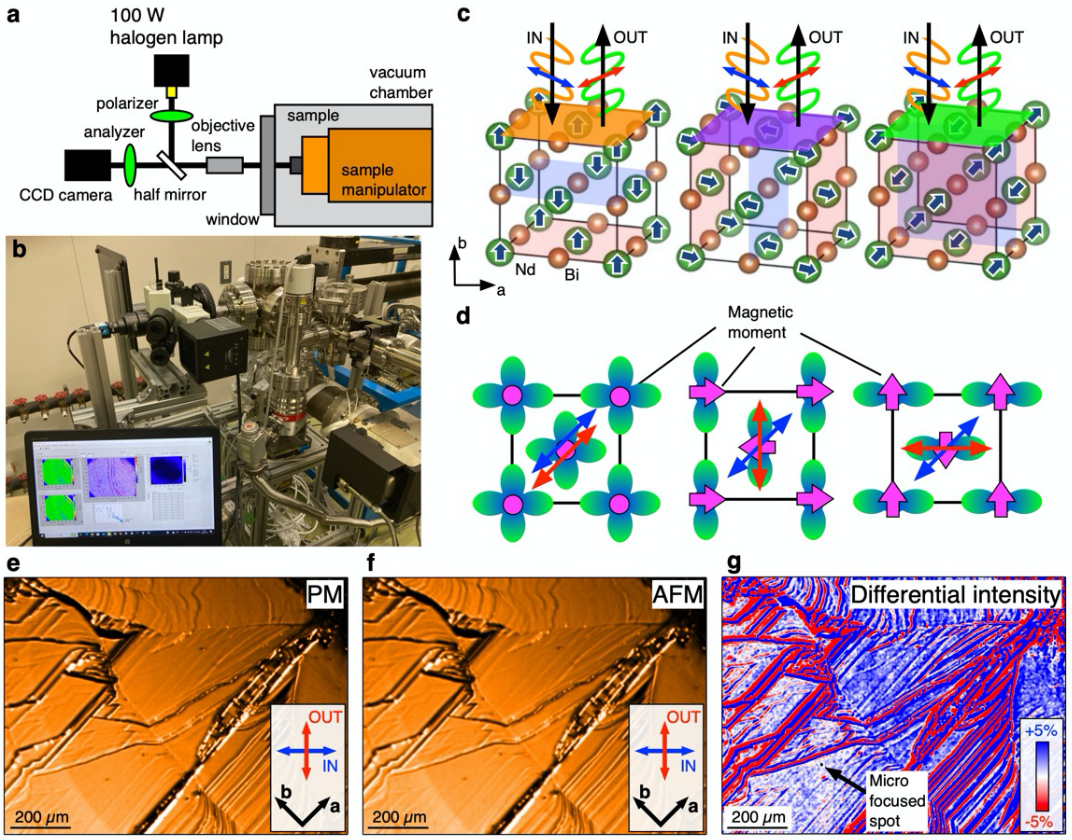

Supplementary Fig. 6 | Polarizing microscopy measurements in the AF phase. **a** Schematic of a constructed polarizing microscopy system. **b** Photograph of the polarizing microscope system. **c** Three types of AF domains in NdBi. **d** Schematics of light polarizations for outgoing photons (red arrows) that have different rotation angles with respect to that of incident photons (blue arrows) depending on the type of AF domains. **e**, **f** Microscope image obtained at $T = 30$ K (PM phase) and 8 K (AF phase), respectively. **g** Polarizing microscope image obtained by subtracting the images between $T = 30$ K and 8 K.

Supplementary Note 6: **Observation of anisotropic electronic states for domain B**

To clarify the symmetry of band dispersion and Fermi surface in the AF phase for domain B, we show in Supplementary Fig. 7a, b the Fermi-surface mapping at $T = 5$ K around the $\bar{\Gamma}$ and $\bar{\mathrm{M}}$ points, respectively. Corresponding ARPES intensity plots as a function of wave vector (k_x or k_y) and binding energy (E_B) obtained along representative **k** cuts (cuts 1–4) are also shown in Supplementary Fig. 7c–f. One can see from the experimental band dispersions along k_x and k_y cuts (cuts 1 and 2) across the $\bar{\Gamma}$ point

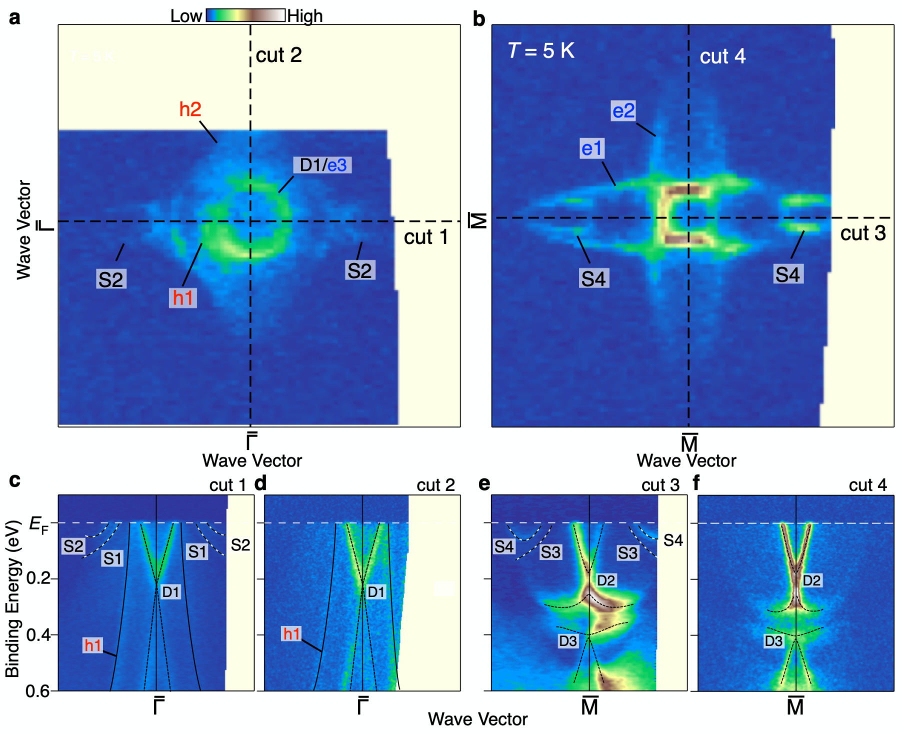

Supplementary Fig. 7 | C_2 symmetric Fermi surface for domain B. a, **b** Fermi-surface mapping around the $\bar{\Gamma}$ and $\bar{M}$ points, respectively, for domain B (same as Fig. 4c, d, respectively). **c**–**f** ARPES intensity in the AF state (T = 5 K) for domain B, measured along four representative **k** cuts (cuts 1–4) shown in **a** and **b**.

(Supplementary Fig. 7c, d) that shallow bands in the vicinity of the Fermi level forming the S2 pocket appear along the k_x cut (cut 1), but not along the k_y cut (cut 2). Similar inequivalence between the vertical and horizontal **k** cuts is also observed around the $\bar{M}$ point, as shown in Supplementary Fig. 7e, f. This demonstrates the C_2 symmetric nature of the band structure and Fermi surface for domain B, supportive of the AF-ordering vector parallel to the in-plane k_x direction, in sharp contrast to the C_4 symmetric Fermi surface for domain A (Fig. 4a, b in the main text).

Supplementary Note 7: Intrinsic nature of the C_2 symmetric electronic states for domain B

We have carried out ARPES measurements with different matrix-element conditions and excluded the matrix-element effect from the origin of the observed C_2 symmetric electronic structure for domain B. Supplementary Fig. 8a, b compare the Fermi surface mapping for domain B obtained with different light polarizations (linear vertical vs circular) and photoelectron emission angles (2nd BZ vs 1st BZ) as shown in the insets. Although the ARPES intensity is strongly modulated by the matrix-element effect, one

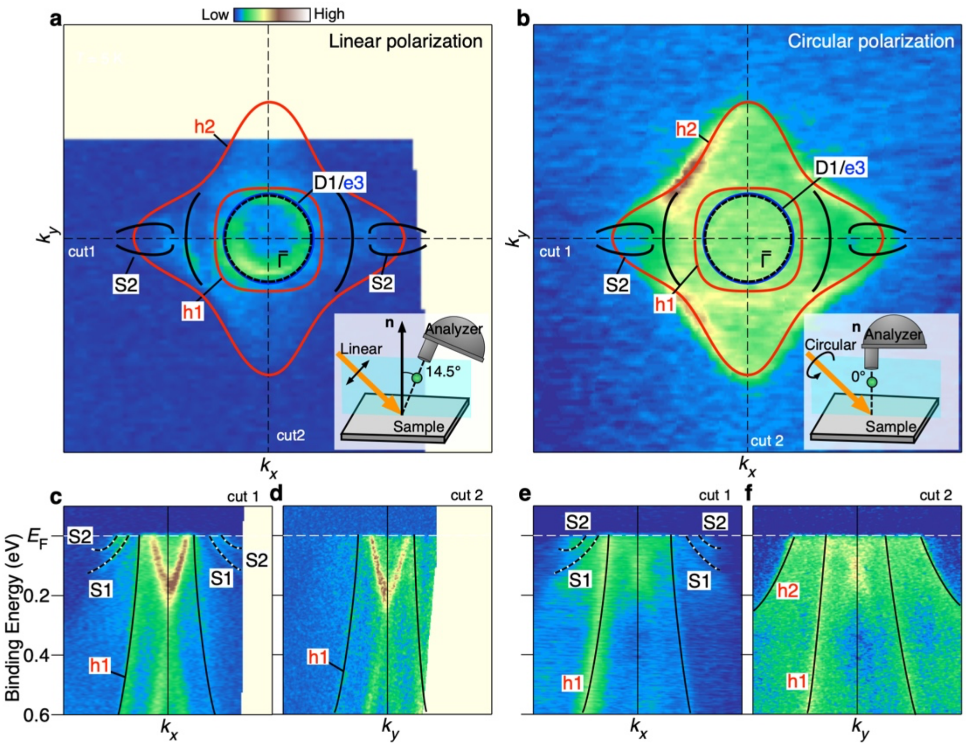

Supplementary Fig. 8 | Light-polarization- and geometry-independent C_2 symmetric nature of Fermi surface for domain B. a ARPES-intensity mapping at E_F as a function of k_x and k_y in the AF phase (T = 5 K) measured with linear vertically polarized light ($h\nu$ = 60 eV) with the measurement geometry shown in the inset which covers the $\bar{\Gamma}$ point of second surface BZ. Black solid curves are a guide for the eyes to trace the experimental Fermi surface. **b** Same as **a** but measured with circularly polarized light with the measurement geometry (inset) which covers the $\bar{\Gamma}$ point of first surface BZ. **c**, **d** ARPES intensity plotted as a function of wave vector (k_x for cut 1 and k_y for cut 2) and E_B. **e**, **f** Same as **c**, **d** but for the measurement geometry of **b**.

can still recognize small S2 pockets along the k_x cut but not along the k_y cut. Such a C_2 symmetric feature is better visualized by the comparison of the ARPES intensity along the k_x and k_y cuts in which the pocket is only seen along the k_x cut irrespective of the difference in the matrix-element effects (Supplementary Fig. 8c–f). This result suggests that the observed C_2 symmetric electronic structure is an intrinsic feature of domain B.

References

[1] Hasegawa, A. Fermi surface of LaSb and LaBi. *J. Phys. Soc. Jpn.* **54**, 677–684 (1985).

[2] Niu, X. H. et al. Presence of exotic electronic surface states in LaBi and LaSb. *Phys. Rev. B* **94**, 165163 (2016).

[3] Nayak, J. et al. Multiple Dirac cones at the surface of the topological metal LaBi. *Nat. Commun.* **8**, 13942 (2017).

[4] Oinuma, H. et al. Unusual change in the Dirac-cone energy band upon a two-step magnetic transition in CeBi. *Phys. Rev. B* **100**, 125122 (2019).

[5] Li, P. et al. Tunable electronic structure and surface states in rare-earth monobismuthides with partially filled f shell. *Phys. Rev. B* **98**, 085103 (2018).

[6] Lou, R. et al. Evidence of topological insulator state in the semimetal LaBi. *Phys. Rev. B* **95**, 115140 (2017).

[7] Wu, Y. et al. Asymmetric mass acquisition in LaBi: Topological semimetal candidate. *Phys. Rev. B* **94**, 081108 (2016).

[8] Duan, X. et al. Tunable electronic structure and topological properties of LnPn (Ln=Ce, Pr, Sm, Gd, Yb; Pn=Sb, Bi). *Commun. Phys.* **1**, 71 (2018).

[9] Kuroda, K. et al. Experimental determination of the topological phase diagram in cerium monopnictides. *Phys. Rev. Lett.* **120**, 086402 (2018).

[10] Kumigashira, H. et al. High-resolution angle-resolved photoemission study of LaSb. *Phys. Rev. B* **58**, 7675–7680 (2002).

[11] Becke, A. D. & Johnson, E. R. A simple effective potential for exchange. *J. Chem. Phys.* **124**, 221101 (2006).

[12] Perdew, J. P., Burke, K. & Ernzerhof, M. Generalized Gradient Approximation Made Simple. *Phys. Rev. Lett.* **77**, 3865–3868 (1996).

[13] Li, P. et al. Tunable electronic structure and surface states in rare-earth monobismuthides with partially filled f shell. *Phys. Rev. B* **98**, 085103 (2018).

[14] Fu, L. & Kane, C. L. Topological insulators with inversion symmetry. *Phys. Rev. B* **76**, 045302 (2007).

[15] Otrokov, M. M. et al. Prediction and observation of an antiferromagnetic topological insulator. *Nature* **576**, 416–422 (2019).

[16] Shikin, A. M. et al. Nature of the Dirac gap modulation and surface magnetic interaction in axion antiferromagnetic topological insulator $MnBi_2Te_4$. *Sci. Rep.* **10**, 13226 (2020).

[17] Lee, S. H. et al. Spin scattering and noncollinear spin structure-induced intrinsic anomalous Hall effect in antiferromagnetic topological insulator $MnBi_2Te_4$. *Phys. Rev. Res.* **1**, 012011(R) (2019).

[18] Schobinger-Papamantellos, P., Fischer, P., Vogt, O. & Kaldis, E. Magnetic ordering of neodymium monopnictides determined by neutron diffraction. *J. Phys. C Solid State Phys*. **6**, 725–737 (1973).

[19] Kuroda, K. et al. Devil's staircase transition of the electronic structures in CeSb. *Nat. Commun.* **11**, 2888 (2020).
