# Supplementary material for: Antiferromagnetic topological insulator with selectively gapped Dirac cones
Source: Nat Commun. 2023 Nov 17;14:7396. doi: 10.1038/s41467-023-42782-6 (PMC10656484; doi:10.1038/s41467-023-42782-6)
Supplement: Supplementary file 2 — Peer Review File [file 41467_2023_42782_MOESM2_ESM.pdf]

nature portfolio

Peer Review File

Antiferromagnetic topological insulator with selectively gapped Dirac cones

Open Access This file is licensed under a Creative Commons Attribution 4.0 International License, which permits use, sharing, adaptation, distribution and reproduction in any medium or format, as long as you give appropriate credit to the original author(s) and the source, provide a link to the Creative Commons license, and indicate if changes were made. In the cases where the authors are anonymous, such as is the case for the reports of anonymous peer reviewers, author attribution should be to 'Anonymous Referee' followed by a clear attribution to the source work. The images or other third party material in this file are included in the article's Creative Commons license, unless indicated otherwise in a credit line to the material. If material is not included in the article's Creative Commons license and your intended use is not permitted by statutory regulation or exceeds the permitted use, you will need to obtain permission directly from the copyright holder. To view a copy of this license, visit http://creativecommons.org/licenses/by/4.0/.

Editorial Note: This manuscript has been previously reviewed at another journal that is not operating a transparent peer review scheme. This document only contains reviewer comments and rebuttal letters for versions considered at *Nature Communications.*

REVIEWERS' COMMENTS

Reviewer #1 (Remarks to the Author):

I read the response to referees and the new version of the manuscript with care, and found that the authors did an excellent job in answering all referee concerns and revising the original paper. In particular, they added several forms of theoretical discussion, including symmetry analysis and DFT calculations, finding that the main claim of the paper is well supported by the results. ARPES data on kz dispersion and temperature variation of the gaps are also enriched. Importantly, the new Figs. 2f and 2k are very important in establishing the fact that these gaps are closed right at the magnetic transition temperature, and thus are likely related to the AFM state. I highly appreciate the substantial work done by the authors to achieve the current form of the paper. I now support the publication of the paper in Nature Communications.
